# Short- and Long-Term Changes in the Network of Parenting Styles and Child Symptoms After a Digital Parent-Training Program: Network Analysis of a Nationwide Implementation Study

**DOI:** 10.2196/92509

**Published:** 2026-11-05

**Authors:** Amit Baumel, Susanna Hinkka-Yli-Salomäki, Yujing Li, Andre Sourander

**Affiliations:** 1 Department of Community Mental Health, University of Haifa, Haifa, Israel; 2 Research Centre for Child Psychiatry, University of Turku, Lemminkäisenkatu 3a, Teutori 3rd Floor, Turku, 20014, Finland, 358 503653447; 3 INVEST Research Flagship Centre, University of Turku, Turku, Finland; 4 Department of Child Psychiatry, Turku University Hospital, Turku, Finland

**Keywords:** network analysis, parent training, digital intervention, child behavior problems, parenting styles

## Abstract

**Background:**

Digital parent-training programs are effective in reducing early childhood behavioral problems, yet little is known about how changes in parenting behaviors relate to sustained changes in child symptoms in real-world, large-scale implementations. Network analysis provides a systems-level framework for examining how parenting styles and child behavioral symptoms dynamically interact and reorganize following digital interventions.

**Objective:**

This study applied network analysis to examine how parenting styles and children’s behavioral symptoms are interrelated before and after a digital-assisted parenting program implemented at a national scale, and how these associations evolve over short- and long-term follow-up (6 and 24 months).

**Methods:**

A total of 36,591 families of 4-year-old children were screened, of whom 4665 (12.7%) met the screening criteria and 2900 were enrolled and received an 11-week digital parenting intervention embedded in Finland’s public health care system. Parent-reported measures of behavioral symptoms and parenting styles were collected at baseline and 6- and 24-month follow-ups. Network analysis was performed using mixed graphical models, and bootstrapping was used to assess changes in edge weights from baseline. Strength centrality indexes were also computed.

**Results:**

At baseline, child aggressive behavior was the most central symptom (strength centrality 1.35), strongly linked to callousness (edge weight 0.45) and emotional reactivity (edge weight 0.34). Parenting variables were peripheral. The associations between changes in parental overreactivity and child aggression were significant (edge weights: 0.18 at 6 months and 0.14 at 24 months; all *P* values <.05). Overreactivity also showed a modest increase in centrality in the change networks (centrality index: 0.75 and 0.73, respectively), while aggressive behavior remained the most central node in the networks of change (centrality index: 1.53 and 1.34, respectively). Several associations among child symptoms, particularly internalizing-related edges, weakened over time.

**Conclusions:**

These findings highlight the value of network analysis for characterizing how the associations between parenting styles and child behavioral symptoms reorganize following a digital parenting program delivered at scale, particularly the strengthened association between changes in overreactive parenting and changes in child aggression. The results support the utility of network analysis for describing durable shifts in behavioral systems and for informing the selection of central intervention targets in future controlled research.

## Introduction

Parent-training interventions are based on the assumption that positive changes in parents’ behaviors will lead to positive changes in children’s behaviors [1-4]. This perspective is grounded in the observation that many parents respond to defiant and oppositional behavior with increased controlling strategies and decreased positive responses [5]. Patterson and Reid [6] described the mechanisms through which this coercive pattern contributes to escalating disruptive and, eventually, more serious antisocial behavior in children. This pattern, which undermines emotional regulation and impairs peer relationships [7-9], has since been confirmed in longitudinal studies [5,10].

Parenting interventions have well-established efficacy in reducing children’s behavioral problems, and change in parenting is theorized to mediate change in child behavior. However, in a systematic review of studies examining parenting as a mediator of intervention outcomes, only a minority of eligible studies reported mediation findings, less than half of the mediation tests performed supported parenting as a mediator, and studies varied considerably in the type of mediation test used, with few establishing that change in parenting preceded change in child outcomes [11,12]. Thus, information about the pathways and mechanisms through which these interventions lead to change remains limited [3,13,14]. Moreover, mediation designs test discrete pathways specified in advance between aggregate constructs, while conventional statistical methods are limited in capturing the dynamic, reciprocal adjustments that unfold across multiple parenting and child domains.

The network approach views mental health problems as emerging from causal relationships among individual symptoms, rather than being driven by a single underlying disorder [15]. According to this view, symptoms can mutually reinforce one another, creating self-sustaining feedback loops. From this perspective, the goal of intervention becomes disrupting these feedback loops by targeting specific symptom-to-symptom connections [16].

In the current study, we employed network analysis to investigate the pathways through which a parenting training program may alter the relationships between parenting styles and children’s behavioral symptoms. Participants were parents of 4-year-old children who received an 11-week web-assisted parenting intervention—the Finnish Strongest Families Parenting (FPSP) program—implemented within routine health care. The FSFP is grounded in the theoretical base common to the family of behavioral parent-training programs, drawing on social learning theory and the coercive family process model [5,6], and it teaches the core components shared by established programs in this family, such as positive attention, praise, and consistent, nonescalating discipline [3,4]. Rather than constituting a new theoretical model, the FSFP is an adaptation of this common approach, based on the model of the Strongest Families program originally developed in Canada [17]. It was culturally adapted for the Finnish context and implemented in both Finnish and Swedish, designed for digital delivery with telephone coaching as part of routine child health services [18,19]. The FSFP intervention was previously evaluated in a randomized clinical trial (October 2011 to November 2013) including 464 families with 4-year-old children who screened positive for disruptive behavior during annual health check-ups; 232 were randomized to the intervention and 232 to an educational control [18]. The findings demonstrated the program’s effectiveness in reducing child behavioral symptoms at 6 and 24 months after randomization [18,20], leading to its adoption in health care settings [21,22].

To our knowledge, no previous studies have examined the network of changes in parenting styles and child behavioral problems following a parent-training program, nor their place within the context of real-world implementation. The first aim of this study was to examine the baseline network of associations between parenting styles and children’s behavioral symptoms and the extent to which parenting behaviors are embedded in this structure. The second and main aim was to examine how the network of changes in parenting and child behaviors unfolds following the intervention, and whether these changes are sustained 2 years after intervention onset. By comparing postintervention networks to the baseline, we aimed to identify meaningful shifts in network structure. This real-world analysis may inform more effective targeting of central nodes and connections within behavioral networks that maintain dysfunction.

## Methods

### Study Design and Participant Recruitment

The present study involved the participation of 2900 families in the FSFP, conducted between January 2015 and December 2021 as part of routine practice at child health clinics across 12 administrative regions of Finland. Upon their child reaching the age of 4, families were provided with an informational package and asked to complete a health questionnaire, which they were instructed to bring to their child’s annual health check-up. During this visit, the FSFP was introduced, and families of children who were deemed eligible were subsequently contacted remotely and were offered the option to enroll in the intervention.

Screening for eligibility in the study was conducted using the conduct problems subscale of the Strengths and Difficulties Questionnaire (SDQ), which was part of the health questionnaire provided to families. A score of 5 or higher, which corresponds to the 80th percentile cutoff based on data from a Finnish study of 4-year-olds [23], was used to identify children exhibiting elevated conduct problems. In addition to meeting the SDQ score criterion, families were required to fulfill the following eligibility criteria: (1) parents reported at least minor concerns regarding the child’s emotional, behavioral, or social functioning; (2) at least one parent was a native speaker of Finnish or Swedish; and (3) the family had access to a phone, computer, and internet connection. The SDQ conduct problems subscale was used for screening because it is a brief measure embedded in the health questionnaire routinely provided to families, which made population-wide screening feasible [24]. The Finnish translation of the Child Behavior Checklist version for Ages 1.5-5 (CBCL/1.5-5), a parent-reported measure of child behavioral and emotional problems [25], and the Inventory of Callous-Unemotional Traits (ICU), which assesses callous and unemotional characteristics in children based on parent reports [26], served as outcome measures because they provide more granular, domain-level assessment of child behavioral and callous-unemotional characteristics suitable for the network analysis, thereby enabling comparison with the original randomized trial of the intervention [18].

### Ethical Considerations

Ethical approval for the implementation study was obtained from the ethics committee of the University of Turku (decisions 44/2014 and 25/2018). Written informed consent was obtained from all participants during enrollment into the study, and they were free to decline participation in the study at any time. Participant privacy was protected through adherence to ethical guidelines, strict data security procedures, and pseudonymizing identifying information. No form of compensation was offered for participating in the study.

### Intervention

The FSFP program combined an interactive web platform with weekly telephone coaching sessions [19]. Coaches, who were professionals from primary health care or social services, including public health nurses and social workers, guided parents through the intervention by conducting weekly phone calls and monitoring their progress via the online platform. The intervention consisted of 11 weekly modules, which were delivered through both the website and personalized telephone coaching.

After completing a baseline questionnaire, parents received an initial phone call from a coach to introduce the program, present the first module, and collaboratively set personalized goals aimed at addressing the child’s behavioral challenges. The first 7 modules focused on developing positive, practical parenting skills, such as constructive problem-solving and understanding emotional development. The subsequent modules emphasized the application and long-term maintenance of these skills in real-life contexts.

Each weekly session included an overview of the topic, instructional materials, video demonstrations, audio guidance, troubleshooting tips, and a summary of key points. Video and audio clips were used to illustrate how to implement the strategies, while the coaches provided tailored feedback and motivation. Progress through the program was structured such that parents could only move on to the next module after successfully completing the previous one. Importantly, children did not participate in the web activities or coaching calls. Consistent with the broader family of behavioral parent-training programs, the modules taught general behavioral parenting skills rather than skills tied to specific parenting style constructs. The conceptual model common to these programs was that acquiring such skills is reflected in broad changes in parenting styles; for example, reduced overreactivity through calm and nonescalating responses to misbehavior and reduced laxness through consistent follow-through on limits [4]. Reductions in dysfunctional parenting styles following behavioral parent training, including digitally delivered programs, have been documented across trials [18,27,28].

### Quality Assurance and Implementation Plan

To ensure data accuracy and maintain the standard of the FSFP, 3 primary quality assurance measures were implemented [19]. First, coaches were carefully selected, provided with comprehensive training, and asked to participate in ongoing supervision to ensure strict adherence to the intervention protocol. Second, continuous quality monitoring was conducted through self-assessments, regular supervision sessions, and the review of a random sample (10%) of recorded phone calls. This process was designed to evaluate coaching competence and ensure consistency across the program.

The implementation of FSFP was coordinated by the Research Centre for Child Psychiatry at the University of Turku. Regular meetings and progress reports were used to support the integration of the program into routine primary health care services. Additionally, public awareness of the program was promoted through local and national media campaigns aimed at encouraging participation and enhancing engagement with the intervention.

### Measures

Child behavioral symptoms were assessed using a Finnish translation of the externalizing scale of the CBCL/1.5-5 [25]. The CBCL/1.5-5 asks parents to rate 99 emotional, behavioral, and social problems and provides an additional section in which they can note extra information on any problem. It yields total scores and syndrome scales for the following items: emotionally reactive, anxious/depressed, somatic complaints, withdrawn, sleep problems, attention problems, and aggressive behavior. The CBCL/1.5-5 demonstrated optimal properties in this study (Cronbach α values were 0.71 for emotionally reactive, 0.70 for anxious/depressed, 0.66 for somatic complaints, 0.62 for withdrawn, 0.71 for sleep problems, 0.65 for attention problems, and 0.86 for aggressive behavior).

The ICU callousness subscale was used to assess deficits in empathy, lack of guilt, and disregard for others’ feelings [26]. A Cronbach α of 0.75 for the callousness subscale was reported in the study population.

The Parenting Scale (PS) is a 30-item questionnaire used to rate participants’ parenting styles based on 3 of its subscales: laxness (5 items), overreactivity (5 items), and hostility (3 items) [29,30]. The Cronbach α values for laxness, overreactivity, and hostility in this study were 0.65, 0.66, and 0.48, respectively. The relatively low internal consistency of the hostility subscale might be partly due to its 3-item structure. Similar findings have been reported previously [30,31].

### Missing Data Handling

Multiple imputation (MI) of missing data was performed using the *multivariate imputation by chained equations* (*mice*) package (version 3.17.0) [32] in R (version 4.4.1; R Foundation for Statistical Computing). The MI approach replaces missing values with multiple sets of reasonable estimates derived from observed data, helping to reduce biases. We specified all the datasets together and used the predictive mean matching (PMM) method to impute the missing values. The PMM method preserves the distributional characteristics of the original data and ensures realistic imputed values. Since the baseline data contained no missing values, they were excluded from the imputation process by setting the imputation method to “empty.” Furthermore, we generated a predictor matrix to define the variables to be used as predictors in each imputation model. To ensure convergence and stability of parameter estimation, we performed the MI using 100 iterations per chain and generated at least 5 imputed datasets. Finally, all the imputed datasets were used for network analysis, and the results were pooled for further analysis.

### Network Analysis

Network analysis was performed using mixed graphical models (MGMs) implemented via the *mgm* package (version 1.2.14) [33] in R. MGMs estimate unique associations (ie, edges) between variables (nodes) within a network by employing an L1-regularized (lasso) nodewise regression approach. The network illustration was made with the package *qgraph* (version 1.9.8) [34] in R. The network models were estimated using data collected before treatment, which served as the study’s baseline data, and then calculated based on changes from baseline to 6- and 24-month follow-up assessments. Children’s CBCL/1.5-5 symptoms and parenting behavior variables were entered into the network analysis as continuous variables [34].

In network analysis, variables are referred to as nodes (for instance, aggression is one node), and the lines connecting the nodes are known as edges. These edges represent associations between the nodes while controlling for the associations between all other nodes in the network [15,35]. Green edges indicate positive associations and red edges indicate negative associations. The edge weight reflects the strength of the relationship between 2 variables, with the width of the edges being proportional to the strength of the association (ie, a thicker edge indicates a stronger relationship) [35].

The change scores (from baseline to 6 or 24 months) were calculated using residualized change scores, a method in which posttest (6- and 24-month) scores are regressed on pretest (baseline) scores, and the difference between the observed posttest scores and the predicted posttest scores is then computed [36,37]. We obtained residualized pre-post change scores by regressing the posttest values of each variable on its corresponding pretest values and then storing the residuals from these regression equations. Residualized change scores were preferred over simple difference scores because they capture the extent to which each family changed beyond what would be expected given its baseline score, thereby reducing the influence of regression to the mean [36,37]. This property matches the aim of the present analysis: the networks were estimated to characterize how changes across parenting and child domains relate to one another in general, rather than to identify which families changed most or benefited most, for which baseline-dependent change would be the quantity of interest. Approaches to modeling change rest on different assumptions and address somewhat different questions [36,37]; residualized scores additionally yield observed continuous variables that can be entered directly into the MGM procedure. When there are a small number of measurement occasions available per family, change-score networks are a noted means of incorporating within-person change, at least to some extent, into network estimation [38].

Strength centrality was estimated as the sum of all absolute edge weights a node was directly connected to. Centrality indexes provide information on which symptoms (ie, nodes) in the network are more strongly connected with other symptoms [39,40]. Higher strength centrality indicates stronger overall connectedness within the estimated network; it does not by itself establish that changes in a central node would produce changes in other symptoms.

To assess the stability of edge weights within the network, we fitted an MGM model using 5000 bootstrap samples. This was conducted using the resample() function of the *mgm* package in R. This procedure generated a distribution of edge weights for each pairwise interaction, enabling the evaluation of edge stability, which was defined as the proportion of bootstrap samples in which each edge retained a nonzero weight. Edges whose bootstrap CIs (95% probability) excluded zero were therefore considered stable and indicative of meaningful associations [33,35]. This method accounted for sampling variability and enhanced confidence in the inferred conditional dependencies, particularly when dealing with mixed variable types [33]. This enabled us to examine whether an edge was significant (ie, its CI did not include zero) and whether the edges in the 6- and 24-month networks—which represent changes from baseline to each respective time point—differed from the corresponding edges at baseline. For brevity, when examining these networks we considered any edge ≤0.10 as practically trivial. This threshold was set a priori to keep the interpretation focused on the strongest associations in the networks. To our knowledge, there is no established convention for interpreting edge magnitudes in network analysis specifically; because the edges in these models are conditional association parameters, we followed the conventional benchmark for a small correlation (*r*=.10) [41-43].

## Results

The implementation process is further detailed in the flowchart presented in Figure 1. A total of 36,591 children were initially screened for symptoms of highly disruptive behavior, of whom 4665 (12.7%) were positive. Among them, 2900 participants were enrolled in the study and completed baseline assessments, of whom 2376 (81.9%) and 2107 (72.7%) completed the 6- and 24-month follow-ups, respectively.

**Figure 1.**
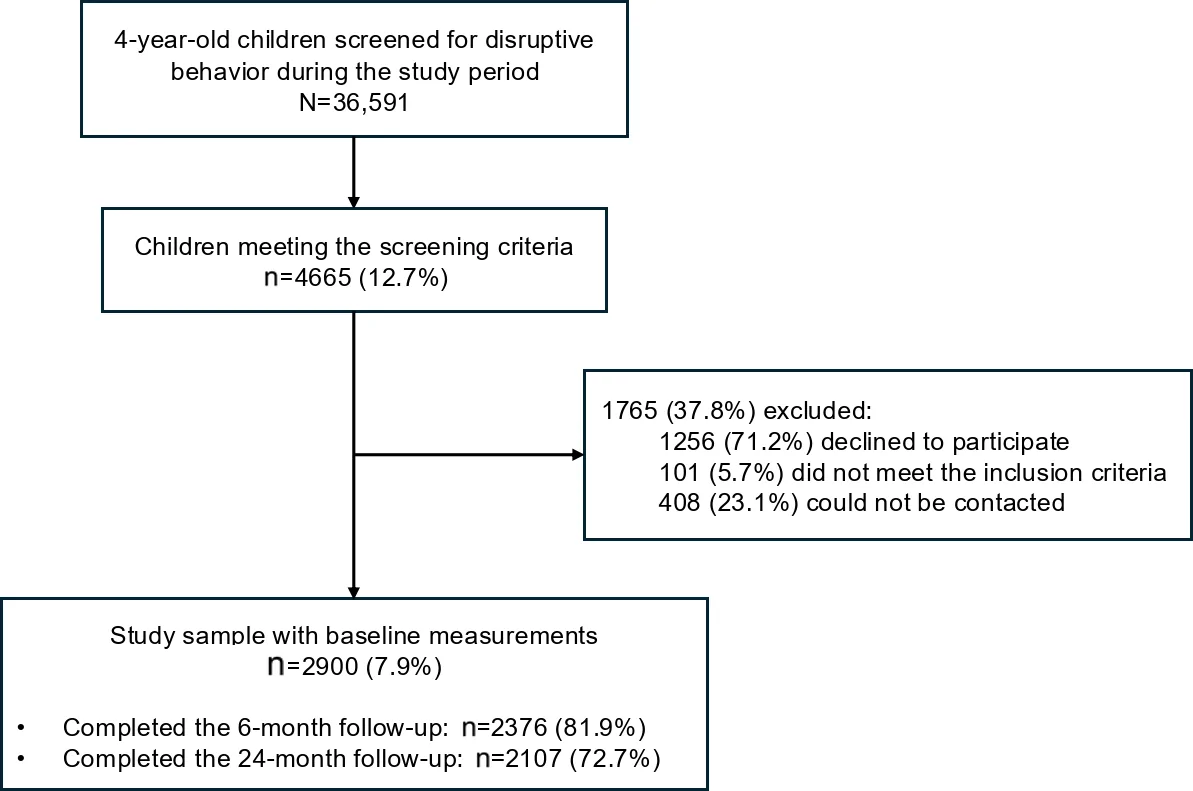
Flow chart of study population selection at different timelines.

Descriptive statistics are presented in Table 1. The sample included 2900 families with children aged 4 years (mean age 4.02, SD 0.10 years), of whom 63.3% (1828/2890) were male. Among parents, 64.1% (1851/2887) of mothers and 47.7% (1302/2729) of fathers held a college or university degree. Participants who did not complete the 24-month follow-up were more likely to have a lower parental educational level, while their baseline child symptoms and parenting practices were similar to those of participants who completed the follow-up (all values for Cohen *d* were ≤0.16; Table S1 in Multimedia Appendix 1).

**Table 1.** Descriptive statistics of demographic and study variables at baseline among 2900 participants.

Variables			Values
Parent and family characteristics			
	Parental age, mean (SD)		
		Maternal age^a^	30.4 (5.0)
		Paternal age^b^	32.6 (5.7)
	Maternal educational level^c^, n (%)		
		Secondary education or less	1036 (35.9)
		College or university degree	1851 (64.1)
	Paternal educational level^d^, n (%)		
		Secondary education or less	1427 (52.3)
		College or university degree	1302 (47.7)
	Enrollment year, n (%)		
		2015‐2017	707 (24.4)
		2018‐2019	1081 (37.3)
		2020‐2021	1112 (38.3)
	Parenting Scale scores, mean (SD)		
		Laxness^e^	2.66 (0.82)
		Overreactivity^f^	3.83 (0.96)
		Hostility^g^	1.81 (0.83)
Child characteristics			
	Sex^h^, n (%)		
		Male	1828 (63.3)
		Female	1062 (36.7)
	Age, mean (SD)		4.02 (0.10)
	Child Behavior Checklist for Ages 1.5-5 scores, mean (SD)		
		Emotionally reactive	4.08 (2.79)
		Anxious/depressed	2.75 (2.34)
		Somatic complaints	2.66 (2.49)
		Withdrawn	2.36 (1.94)
		Sleep problems	4.34 (2.82)
		Attention problems	3.26 (1.87)
		Aggressive behavior	18.46 (6.29)
	Inventory of Callous-Unemotional Traits score, mean (SD)		
		Callousness^i^	8.70 (4.13)

a Missing: n=23.

b Missing: n=129.

c Missing: n=13.

d Missing: n=171.

e Missing: n=1.

f Missing: n=1.

g Mssing: n=1.

h Missing: n=10.

i Missing: n=1.

The baseline network structure and the networks of change at the 6- and 24-month follow-ups are presented in Figure 2A-C, with their corresponding strength centrality indexes shown in Figure 3A-C. At baseline, child aggressive behavior (centrality index 1.35), emotionally reactive (centrality index 1.33), anxious/depressed (centrality index 1.27), and callousness (centrality index 1.21) were the most central nodes. Aggressive behavior showed moderate associations with emotionally reactive (edge weight 0.34) and callousness (edge weight 0.45), while emotionally reactive was linked to anxious/depressed (edge weight 0.46). Parenting variables were peripheral at baseline, though overreactivity showed a moderate association with parental hostility (edge weight 0.40).

**Figure 2.**
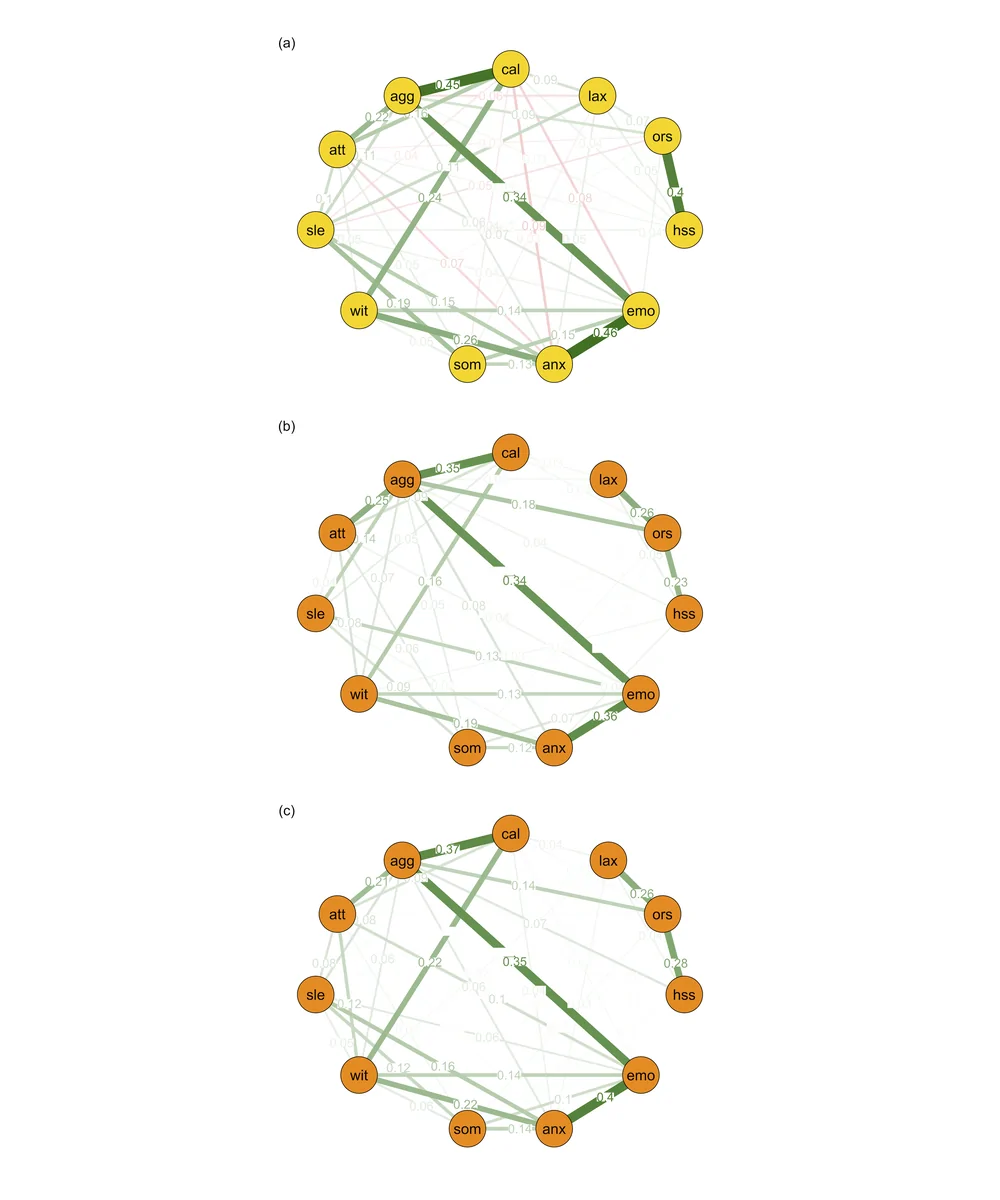
Network representations of parenting and child variables. Thicker edges indicate stronger associations between the nodes. Green edges indicate positive associations and red edges indicate negative associations. The numerical values on the edges represent the strength of these connections after accounting for all other node connections in the network. (A) Baseline network. (B) Network of changes from baseline to 6 months. (C) Network of changes from baseline to 24 months. Agg: aggressive behavior; anx: anxious/depressed; att: attention problems; cal: callousness; emo: emotionally reactive; hss: hostility; lax: laxness; ors: overreactivity; sle: sleep problems; som: somatic complaints; wit: withdrawn.

**Figure 3.**
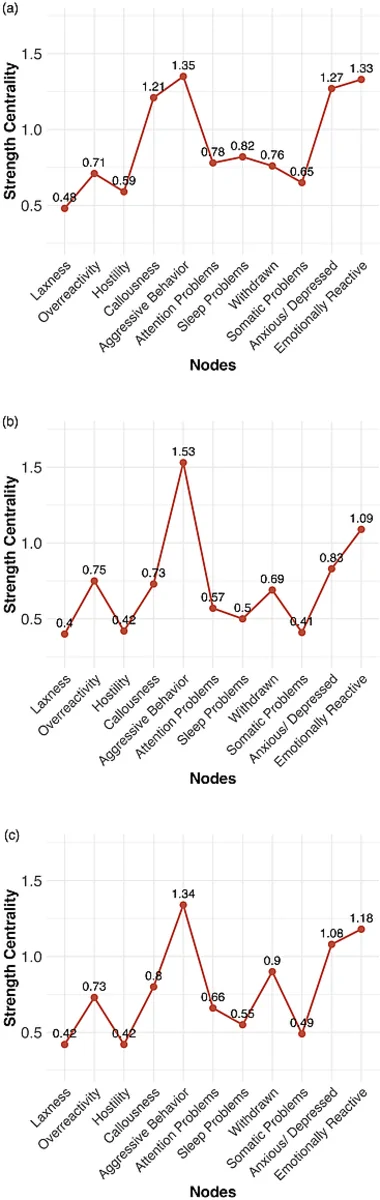
Strength centrality of changes from baseline to 24 months. (A) Strength centrality at baseline. (B) Strength centrality of changes from baseline to 6 months. (C) Strength centrality of changes from baseline to 24 months.

Compared to baseline, strength centrality for aggressive behavior was higher in the 6-month change network (centrality index 1.53) and similar in the 24-month change network (centrality index 1.34), while most other child symptom associations weakened. Centrality of parenting variables showed small shifts, with a slight increase for overreactivity (centrality index 0.75 at 6 months and 0.73 at 24 months), whereas hostility and laxness remained comparatively low.

Table 2 presents bootstrap estimates and CIs of the networks’ edges. Notably, the association between changes in parental overreactivity and changes in child aggressive behavior increased significantly from baseline (bootstrap mean 0.08) to 6 months (bootstrap mean 0.18) and remained higher at 24 months (bootstrap mean 0.14). A stronger association was also observed between changes in overreactivity and laxness (bootstrap mean increased from 0.08 at baseline to 0.26 at 6 and 24 months), while the link between overreactivity and hostility decreased (bootstrap mean decreased from 0.40 at baseline to 0.23 and 0.28 at 6 and 24 months, respectively). Within the child domain, 3 edges showed sustained reductions: aggressive and callousness (bootstrap mean decreased from 0.45 at baseline to 0.35 and 0.37 at 6 and 24 months), anxious/depressed and emotionally reactive (bootstrap mean decreased from 0.46 at baseline to 0.36 and 0.40), and anxious/depressed and withdrawn (bootstrap mean decreased from 0.26 at baseline to 0.19 and 0.22).

**Table 2.** Bootstrapped estimation for network edges. Italics indicate edges in the 6- or 24-month change networks that significantly differed from the corresponding edges at baseline and edge >0.10 at that time point.

Edges		Network edge estimates		
Node 1	Node 2	Bootstrap mean at baseline (95% CI)	Bootstrap mean at 6 months (95% CI)	Bootstrap mean at 24 months (95% CI)
Laxness	Overreactivity	0.08 (0.04 to 0.11)	*0.26 (0.24 to 0.28)*	*0.26 (0.25 to 0.28)*
Laxness	Anxious/depressed	0.05 (0.00 to 0.09)	–0.00 (–0.02 to 0.01)	0.04 (0.02 to 0.05)
Laxness	Sleep problems	0.11 (0.08 to 0.15)	0.01 (–0.00 to 0.02)	0.02 (0.00 to 0.03)
Laxness	Aggressive behavior	–0.07 (–0.11 to –0.03)	0.03 (0.01 to 0.05)	–0.01 (–0.02 to 0.01)
Laxness	Hostility	0.05 (0.02 to 0.09)	0.06 (0.04 to 0.07)	0.05 (0.03 to 0.07)
Overreactivity	Hostility	0.40 (0.36 to 0.43)	*0.23 (0.21 to 0.25)*	*0.28 (0.26 to 0.30)*
Overreactivity	Sleep problems	–0.05 (–0.09 to 0.00)	0.01 (–0.00 to 0.02)	0.00 (–0.01 to 0.02)
Overreactivity	Aggressive behavior	0.08 (0.04 to 0.12)	*0.18 (0.16 to 0.19)*	*0.14 (0.12 to 0.15)*
Hostility	Attention problems	0.03 (0.00 to 0.07)	0.04 (0.02 to 0.06)	0.07 (0.05 to 0.09)
Callousness	Laxness	0.10 (0.06 to 0.13)	0.03 (0.01 to 0.05)	0.04 (0.02 to 0.06)
Callousness	Emotionally reactive	–0.08 (–0.12 to –0.04)	0.01 (–0.00 to 0.03)	–0.02 (–0.04 to –0.00)
Callousness	Anxious/depressed	–0.09 (–0.13 to –0.05)	0.00 (–0.01 to 0.01)	–0.04 (–0.06 to –0.03)
Callousness	Withdrawn	0.23 (0.20 to 0.27)	*0.16 (0.15 to 0.18)*	0.22 (0.21 to 0.24)
Callousness	Attention problems	0.16 (0.12 to 0.19)	0.09 (0.07 to 0.10)	0.09 (0.08 to 0.11)
Callousness	Aggressive behavior	0.45 (0.41 to 0.47)	*0.35 (0.34 to 0.37)*	*0.37 (0.35 to 0.39)*
Callousness	Sleep problems	–0.04 (–0.08 to 0.00)	0.05 (0.04 to 0.07)	0.00 (–0.01 to 0.02)
Attention problems	Aggressive behavior	0.22 (0.19 to 0.26)	*0.25 (0.23 to 0.26)*	0.22 (0.20 to 0.23)
Sleep problems	Attention problems	0.10 (0.06 to 0.14)	0.04 (0.03 to 0.06)	0.08 (0.06 to 0.10)
Sleep problems	Aggressive behavior	0.11 (0.07 to 0.15)	*0.14 (0.12 to 0.15)*	0.08 (0.06 to 0.09)
Withdrawn	Attention problems	0.05 (0.02 to 0.09)	0.08 (0.07 to 0.10)	*0.12 (0.10 to 0.14)*
Withdrawn	Sleep problems	–0.00 (–0.04 to 0.03)	–0.01 (–0.02 to 0.01)	0.05 (0.04 to 0.07)
Withdrawn	Aggressive behavior	–0.00 (–0.04 to 0.03)	0.07 (0.06 to 0.09)	0.06 (0.05 to 0.08)
Somatic complaints	Sleep problems	0.19 (0.15 to 0.22)	0.09 (0.08 to 0.11)	*0.12 (0.10 to 0.14)*
Somatic complaints	Attention problems	0.05 (0.00 to 0.09)	0.05 (0.04 to 0.07)	0.03 (0.02 to 0.05)
Somatic complaints	Withdrawn	0.05 (0.00 to 0.09)	0.01 (–0.00 to 0.03)	0.06 (0.04 to 0.08)
Anxious/depressed	Somatic complaints	0.13 (0.09 to 0.17)	0.12 (0.10 to 0.14)	0.14 (0.12 to 0.16)
Anxious/depressed	Withdrawn	0.26 (0.22 to 0.30)	*0.19 (0.17 to 0.21)*	*0.22 (0.21 to 0.24)*
Anxious/depressed	Sleep problems	0.15 (0.11 to 0.18)	0.03 (0.01 to 0.05)	0.16 (0.14 to 0.18)
Anxious/depressed	Attention problems	–0.07 (–0.11 to –0.03)	0.01 (–0.00 to 0.02)	–0.01 (–0.03 to 0.00)
Anxious/depressed	Aggressive behavior	0.06 (0.02 to 0.09)	0.08 (0.06 to 0.10)	0.06 (0.04 to 0.08)
Emotionally reactive	Anxious/depressed	0.46 (0.43 to 0.50)	*0.36 (0.35 to 0.38)*	*0.40 (0.38 to 0.42)*
Emotionally reactive	Somatic complaints	0.15 (0.11 to 0.19)	0.07 (0.05 to 0.09)	0.10 (0.08 to 0.12)
Emotionally reactive	Withdrawn	0.15 (0.10 to 0.19)	*0.13 (0.11 to 0.14)*	0.14 (0.12 to 0.15)
Emotionally reactive	Attention problems	0.08 (0.03 to 0.11)	0.04 (0.02 to 0.06)	0.10 (0.09 to 0.12)
Emotionally reactive	Sleep problems	0.04 (0.00 to 0.08)	*0.13 (0.11 to 0.15)*	0.06 (0.03 to 0.08)
Emotionally reactive	Aggressive behavior	0.34 (0.31 to 0.37)	0.34 (0.33 to 0.36)	0.35 (0.34 to 0.37)

We conducted the same network analysis and strength centrality calculations for the complete samples without any missing observations, and the pattern of results (ie, the network topology, edge weights, and strength centrality) was similar (Figures S1-S4 in Multimedia Appendix 1), suggesting the findings were robust to the handling of missing data.

## Discussion

To the best of our knowledge, this is the first study to examine the network of changes in parenting and child behaviors following a parent-training program, as well as the sustained pattern of changes observed over a 2-year period in an implementation setting that, for research on digital parenting interventions, had a large sample (2900 families). These real-world care intervention study results are, therefore, key to our understanding of how changes in parenting styles relate to changes in child behaviors.

At baseline, the network structure of children’s behavioral symptoms revealed a densely connected externalizing cluster centered on aggressive behavior. Aggressive behavior demonstrated significant associations with both callousness (edge weight 0.45) and attention problems (edge weight 0.22), consistent with established models linking callous-unemotional traits and attentional deficits to early conduct problems (eg, Frick and Morris [44] and Waller et al [45]). Aggressive behavior was also associated with emotional reactivity (edge weight 0.34), which in turn was linked to anxious/depressed (edge weight 0.46), which was linked to withdrawal symptoms (edge weight 0.26). This internalizing pathway may represent an internalizing subnetwork that interacts with externalizing problems. Internalizing pathways may contribute to the maintenance or escalation of aggression in some children through emotional dysregulation [46]. The co-occurrence of these pathways suggests that for some children, aggressive behavior may arise not only from trait-like dispositions but also from heightened emotional sensitivity.

Overall, the baseline network highlights child aggression as a key organizing feature of behavioral dysregulation in early childhood, embedded in a structure where callous traits, attention problems, and emotional reactivity form tightly interwoven feedback loops. Interestingly, at baseline, parenting styles showed only weak associations with children’s behavioral symptoms, despite the entire sample consisting of families with children identified as having behavior problems. This may reflect either genuinely weak associations before the intervention or restricted variability resulting from the screening procedure. Of the two possibilities, restricted range appears the more plausible contributor: selection for an elevated SDQ score for conduct problems directly restricted the screening measure and indirectly restricted the range of correlated variables, particularly the child symptom measures, attenuating the associations that can be observed between them [47].

To address the second and primary aim of the study, we examined how the network of associations between parenting styles and children’s behavioral symptoms changed following the intervention and whether these changes were sustained over time. Compared to the baseline network, which showed only limited connections between parenting and child symptoms, the network of change at 6 months revealed notable shifts, particularly in edges involving parenting variables. The most important of these was the emergence of a stronger connection between parental overreactivity and child aggression, which, when compared with baseline (edge weight 0.08), became significantly stronger at both 6 months (edge weight 0.18) and 24 months (edge weight 0.14). Among parents who reported reduced overreactive responses following the intervention, a parallel reduction in child aggression was observed—aligning with prior findings showing co-occurring changes in parenting styles and child conduct problems [27,28]. Although the strength centrality of overreactivity increased somewhat in the postintervention change networks, parenting variables did not become highly central; rather, aggressive behavior remained the dominant hub of the system.

Beyond this cross-domain shift, we also observed some restructuring within parenting behaviors themselves. The edge between overreactivity and laxness increased markedly (edge weights: 0.08 at baseline; 0.26 at 6 months; 0.26 at 24 months). In contrast, the edge between overreactivity and hostility decreased over time (edge weights: 0.40 at baseline; 0.23 at 6 months; 0.28 at 24 months). Taken together, these findings reflect a more nuanced pattern of intraparenting change: while overreactivity became more closely linked with laxness, its association with hostility weakened. Notably, given the relatively low internal consistency of the hostility subscale, findings should be interpreted cautiously. One possible interpretation is that hostility represents a more trait-like, dispositional form of harsh parenting closely tied to chronic parental anger and negative affect [48], which could be less responsive to brief behavioral interventions. In contrast, overreactivity and laxness reflect more state-like, interaction-based discipline processes that co-vary during parent-child exchanges and therefore show coordinated change. These interpretations are post hoc and should be treated as hypotheses rather than conclusions. An alternative explanation is that discipline practices become temporarily less consistent during skill acquisition, as parents disrupt previous response patterns before new ones have stabilized, which would produce correlated fluctuation across overreactivity and laxness. However, the persistence of the strengthened association at 24 months argues against a purely temporary destabilization account. Future studies with denser measurement of parenting practices are needed to distinguish between these accounts.

At the same time, several associations among child symptoms weakened in the networks of change. The links between changes in aggression and callousness, anxious/depressed, and emotionally reactive behavior, as well as between changes in anxious/depressed and withdrawn behavior, were all smaller relative to associations at baseline. The intervention did not directly target internalizing symptoms. In interpreting this pattern, the change networks capture the coupling of changes rather than of symptom levels: a weaker edge indicates that changes in these symptoms were less strongly intercorrelated, not that the symptoms themselves became unrelated. As the intervention targeted child externalizing behavior through parenting, coordinated change would be expected to concentrate in that domain, whereas internalizing symptoms may have changed in a less coordinated manner. These are secondary findings that should be interpreted with caution and examined in future studies. Moreover, these changes were modest in magnitude, and aggressive behavior remained the most central node in both change networks. Accordingly, the findings suggest more targeted shifts, particularly in the relationship between overreactive parenting and child aggression, rather than a broad reorganization of the behavioral network or a substantial increase in the centrality of parenting variables.

This study has several limitations. First, this was a single-arm implementation study without a control condition. The observed changes in the networks cannot be attributed to the intervention, as natural maturation, regression to the mean, and other time-related influences cannot be ruled out. In this design, network analysis is descriptive: it characterizes how the structure of associations changed following the intervention, but it cannot determine why it changed. Furthermore, network analysis does not directly examine whether a change in one variable precedes a change in another; hence, it is not built to establish causal associations. The intervention was previously shown to reduce child behavioral symptoms relative to an educational control at the same follow-up points in a randomized clinical trial [18,20], and child and parenting outcomes of the nationwide implementation did not differ significantly from the randomized clinical trial intervention arm [22], which supports the plausibility of intervention-related change, although the present design cannot test it. Second, although the study used a large, real-world sample and a longitudinal design, all outcome measures were based solely on parent-reported questionnaires, which may have introduced reporting bias and limited the objectivity of symptom assessments. Shared-method variance is a particular concern for the network estimates, as correlated measurement error between scales completed by the same informant can inflate, or even produce, edges between nodes. Cross-domain edges between parenting and child variables should be interpreted with this in mind, and future studies would benefit from multi-informant assessment, such as teacher reports or observational measures. Third, the generalizability of the findings may be limited by the sample’s cultural and health care context, drawn entirely from Finland’s universal health care system, which may not reflect conditions in countries with different systems of parental support or different levels of access to digital interventions. In addition, eligibility required that at least one parent was a native speaker of Finnish or Swedish and that the family had access to a phone, a computer, and an internet connection. Internet access is close to universal among Finnish households with children (more than 95% [49]), so digital exclusion was minimal in this context, although the reach of digital interventions may differ in countries with less extensive digital infrastructure. Moreover, because families lost to the 24-month follow-up tended to have a lower parental educational level, the observed associations may partly reflect selective attrition toward more socioeconomically advantaged families in addition to longitudinal changes.

Nonetheless, the network approach is a step toward a paradigm shift in how we conceptualize and study mental health and behavioral interventions. Rather than viewing symptoms as isolated or as mere manifestations of an underlying disorder, this approach considers the dynamic interplay among symptoms and behaviors within complex systems. It enables us to examine long-standing and clinically meaningful questions, such as how interventions work, when, and for whom [1,3,14,50]. By illuminating which changes in parenting are most central to broader shifts in child behavior—and how these changes cluster or unfold over time—network analysis opens new avenues for precision intervention. Ultimately, this approach could enhance our understanding of the mechanisms underlying behavior change and inform the design of more effective, targeted, and sustainable interventions tailored to individual family needs.

## Supplementary material

10.2196/92509 Multimedia Appendix 1 Comparisons of baseline demographics, parenting styles, and child symptoms by 24-month follow-up completion status (Table S1), as well as networks of residualized changes and corresponding strength centrality at 6 and 24 months based on complete-case analyses (Figures S1-S4).

## Abbreviations

CBCL/1.5-5: Child Behavior Checklist for Ages 1.5-5
FSFP: Finnish Strongest Families Parenting
ICU: Inventory of Callous-Unemotional Traits
MGM: mixed graphical model
MI: multiple imputation
mice: multivariate imputation by chained equations
PMM: predictive mean matching
PS: Parenting Scale
SDQ: Strengths and Difficulties Questionnaire
