# Supplementary material for: Short- and Long-Term Changes in the Network of Parenting Styles and Child Symptoms After a Digital Parent-Training Program: Network Analysis of a Nationwide Implementation Study
Source: JMIR Pediatr Parent. 2026 Nov 5;9:e92509. doi: 10.2196/92509 (PMC13739263; doi:10.2196/92509)
Supplement: Supplementary file 1 [file pediatrics-v9-e92509-s001.docx]

**SUPPLEMENT**

**Table S1.** Demographic characteristics of participants by 24-month follow-up completion status

| **Demographics** | **Completed 24-month follow-up (N = 2,107)** | **Did not complete 24-month follow-up (N = 793)** | **p-value *^j^*** | **Effect size (Cohen’s *d*/Odds Ratio/** **Cramer’s *V*) ^k^** |
| --- | --- | --- | --- | --- |
| **Parent and family characteristics** | | | |  |
| Parental age, mean (SD) | | | |  |
| Maternal ^a^ | 30.7 (4.9) | 29.8 (5.3) | <.001 | 0.18 |
| Paternal ^b^ | 32.8 (5.5) | 32.1 (6.1) | .017 | 0.12 |
| Maternal educational level ^c^, n (%) | | | |  |
| Secondary education or less | 655 (31.2) | 381 (48.5) | <.001 | 2.08 |
| College or university degree | 1,446 (68.8) | 405 (51.5) |  |  |
| Paternal educational level ^d^, n (%) | | | |  |
| Secondary education or less | 980 (49.0) | 447 (61.4) | <.001 | 1.66 |
| College or university degree | 1,021 (51.0) | 281 (38.6) |  |  |
| Enrollment year, n (%) |  |  |  |  |
| 2015-2017 | 510 (24.2) | 197 (24.8) | <.001 | 0.08 |
| 2018-2019 | 833 (39.5) | 248 (31.3) |  |  |
| 2020-2021 | 764 (36.3) | 348 (43.9) |  |  |
| Parenting Scale scores, mean (SD) |  |  |  |  |
| Laxness ^e^ | 2.7 (0.8) | 2.7 (0.9) | .313 | 0.00 |
| Overreactivity ^f^ | 3.9 (1.0) | 3.8 (1.0) | .053 | 0.10 |
| Hostility ^g^ | 1.8 (0.8) | 1.9 (0.8) | <.001 | 0.13 |
| **Child characteristics** |  |  |  |  |
| Sex ^h^, n (%) | | | |  |
| Male | 1,323 (63.0) | 505 (64.0) | .607 | 1.05 |
| Female | 778 (37.0) | 284 (36.0) |  |  |
| Age, mean (SD) | 4.0 (0.1) | 4.0 (0.1) | .008 | 0.00 |
| CBCL scores, mean (SD) |  |  |  |  |
| Emotionally reactive | 4.0 (2.7) | 4.3 (2.9) | .033 | 0.11 |
| Anxious/Depressed | 2.7 (2.3) | 3.0 (2.4) | <.001 | 0.13 |
| Somatic complaints | 2.6 (2.5) | 2.8 (2.5) | .136 | 0.08 |
| Withdrawn | 2.3 (1.9) | 2.5 (2.0) | .028 | 0.10 |
| Sleep problems | 4.3 (2.8) | 4.4 (2.9) | .317 | 0.03 |
| Attention problems | 3.2 (1.8) | 3.5 (1.9) | <.001 | 0.16 |
| Aggressive behavior | 18.3 (6.2) | 19.0 (6.5) | .003 | 0.11 |
| Callousness score ^i^, mean (SD) | 8.6 (4.1) | 8.9 (4.2) | .355 | 0.07 |

^a^ Missing values: completed the follow-up n = 12, did not complete the follow-up n = 11;

^b^ Missing values completed the follow-up n = 78, did not complete the follow-up n = 51;

^c^ Missing values: completed the follow-up n = 6, did not complete the follow-up n = 7;

^d^ Missing values: completed the follow-up n = 106, did not complete the follow-up n = 65;

^e^ Missing values: did not complete the follow-up n = 1;

^f^ Missing values: did not complete the follow-up n = 1;

^g^ Missing values: did not complete the follow-up n = 1;

^h^ Missing values: completed the follow-up n = 6, did not complete the follow-up n = 4;

^i^ Missing values: did not complete the follow-up n = 1.

^j^ Wilcoxon rank sum test for continuous variables; Pearson’s Chi-squared test for categorical variables.

^k^ Reported as Cohen’s *d* for continuous variables, odds ratios (ORs) for binary categorical variables, and Cramer’s *V* for variables with more than two categories. For ORs, the first category listed was used as the reference group.

SD = standard deviation; CBCL = Child Behavior Checklist 1.5-5.

**Figure S1.** Network representations of residualized changes in parenting and child variables from baseline to the 6-month follow-up using complete cases. Thicker edges indicate stronger associations between the nodes. Green edges indicate positive associations and red edges indicate negative relationships. The numerical values on the edges represent the strength of these connections after accounting for all other nodes connections in the network. Abbreviations: agg = aggressive behavior; anx = anxious/depressed; att = attention problems; cal = callousness; emo = emotionally reactive; hss = hostility; lax = laxness; ors = overreactivity; sle = sleep problems; som = somatic complaints; wit = withdrawn.
**Figure S2.** Network representations of residualized changes in parenting and child variables from baseline to the 24-month follow-up using complete cases. Thicker edges indicate stronger associations between the nodes. Green edges indicate positive associations and red edges indicate negative relationships. The numerical values on the edges represent the strength of these connections after accounting for all other nodes connections in the network. Abbreviations: agg = aggressive behavior; anx = anxious/depressed; att = attention problems; cal = callousness; emo = emotionally reactive; hss = hostility; lax = laxness; ors = overreactivity; sle = sleep problems; som = somatic complaints; wit = withdrawn.

**Figure S3.** Strength centrality of changes from baseline to 6 months with complete cases.

**Figure S4.** Strength centrality of changes from baseline to 24 months with complete cases.
